# Addressing cancer prevention and control in Armenia: tobacco control and mHealth as key strategies

**DOI:** 10.1186/s12939-020-01344-8

**Published:** 2021-01-06

**Authors:** Carla J. Berg, Arusyak Harutyunyan, Nino Paichadze, Adnan A. Hyder, Varduhi Petrosyan

**Affiliations:** 1 Department of Prevention and Community Health, Milken Institute School of Public Health, George Washington Cancer Center, George Washington University, Washington, DC, USA; 2 Turpanjian School of Public Health, American University of Armenia, Yerevan, Armenia; 3 Center on Commercial Determinants of Health and Department of Global Health, Milken Institute School of Public Health, Washington, DC, USA

**Keywords:** Cancer prevention, Cancer control, Tobacco control, Policy, mHealth

## Abstract

This paper focuses on the particular challenges in cancer prevention and control (CPC) in low- and middle-income countries (LMICs). In particular, this paper extrapolates challenges and opportunities in Armenia, which has the 2nd highest rate of cancer-related deaths in the world, the 11th highest smoking prevalence among men globally, and an evolving health system infrastructure for non-communicable disease (NCD) prevention and control, including CPC. Despite significant progress in enhancing research capacity in Armenia over the past decade, additional efforts are needed, particularly in CPC-related research. Key opportunities are to advance tobacco control and utilization of mHealth. Public health training programs remain insufficient in the area of CPC, and in-country research expertise regarding CPC and related areas (e.g., tobacco control, mHealth, policy) is limited, particularly given the need to address the diverse and complex determinants of onset, prevention, and management of cancer. Moreover, critical gaps in research dissemination and knowledge translation from evidence to policy and practice continue to exist. Thus, public health infrastructure must be enhanced, in-country CPC leaders across various relevant disciplines must be further developed and supported, and medical and public health training must more fully integrate CPC and research dissemination and translation to inform policy and practice.

## Overview

This paper focuses on challenges in cancer prevention and control (CPC) in low- and middle-income countries (LMICs), particularly in Armenia, which has the 2nd highest rate of cancer-related deaths in the world, the 11th highest smoking prevalence among men globally, and evolving health system infrastructure for non-communicable disease (NCD) prevention and control, including CPC [1–3].

## Global impact of cancer

Cancer is the second leading cause of death globally, causing ~ 1 in 6 deaths [4]. The most common cancers are lung, breast, colorectal, prostate, skin (non-melanoma), and stomach, with several of these also causing the most cancer-related deaths (e.g., lung, colorectal, stomach, breast) [4]. Based on population growth and aging, the global cancer burden will grow to 29.4 M cases annually in 2040 [4].

LMICs account for ~ 70% of cancer-related deaths [4] and, by 2030, will account for 75% of new cancer cases and deaths [4]. Although several countries have achieved significant cancer burden reductions, disparities in progress exist [4]. While incidence of preventable malignancies (e.g., lung) has decreased in high-income countries (HICs) but has not changed or increased in LMICs [4].

CPC is a critical component in reducing cancer burden and disparities. Five behavioral risks (tobacco use, alcohol use, overweight/obesity, low fruit/vegetable intake, low physical activity) contribute to ~ 1/3 of cancer deaths, 30–50% of which are preventable by avoiding risk factors and implementing evidence-based prevention strategies [4]. Additionally, early detection and treatment can reduce cancer mortality [4]. However, late-stage presentation and inaccessible diagnosis and treatment are prominent in LMICs [4].

## Global impact of tobacco

The tobacco epidemic is among the biggest global health threats, with > 7 M tobacco-related deaths per year [5] with estimated increases to > 8 M per year by 2030 [5]. Tobacco use is among the most important cancer risk factors – responsible for ~ 22% of cancer deaths (and other chronic diseases, e.g., cardiovascular diseases) [5]. Nonsmokers are also impacted via secondhand smoke exposure (SHSe); ~ 1.8B nonsmokers, including 40% of children and ~ 34% of adult nonsmokers experience SHSe, estimated to kill > 600,000 annually [6]. Unfortunately, only 7% of the world lives in places with comprehensive smoke-free laws [6].

Tobacco-related morbidity and mortality is increasingly burdening LMICs [5]; 80% of the 933 M current daily smokers [5] live in LMICs [5], half of whom will die prematurely due to smoking [5]. The disproportionate impact of tobacco use is also reflected in the impact of SHSe [6].

## Global action to address CPC & tobacco

In 2017, the World Health Assembly passed the *Cancer Prevention and Control through an Integrated Approach* to catalyze acheiving specific targets in the *Global Action Plan* and *2030 UN Agenda for Sustainable Development* (to reduce premature cancer mortality). Key priorities include surveillance, CPC research and translation, identifying cost-effective priority CPC strategies, and developing standards/tools to guide interventions and health systems improvements to address cancer across the cancer continuum.

Additionally, for over a decade, the World Health Organization (WHO) Framework Convention on Tobacco Control (FCTC) has promoted adoption of comprehensive evidence-based policies (e.g., taxation, smoke-free air) to counter the tobacco epidemic; 181 countries have ratified the FCTC (covering ~ 90% of the world’s population), most of which are LMICs [7].

## mHealth interventions in LMICs

Identifying solutions to address cancer burden in LMICs with wide reach, strong potential for scale-up, and the ability to strengthen existing health systems is critical. mHealth (i.e., mobile/wireless devices to improve health) has shown utility in increasing healthcare access, quality, effectiveness, and cost-effectiveness [8]. The potential utility and impact of mHealth in LMICs is particularly great, given the popularity and availability of mobile devices across demographics and contexts and given that access to mobile phones is often greater than access to regular healthcare in many LMICs [8].

However, mHealth technology adoption is higher in HICs versus LMICs [8], perhaps due to better understanding, skills, and resources to build and implement mHealth, not the least of which are human capacities/understanding [8]. In LMICs, most health systems are overstrained and face ongoing challenges to make complex decisions about competing priorities [8]. Thus, promoting mHealth as one relevant, potentially high-impact solution to healthcare challenges is critical in addressing cancer and general NCD burden in LMICs [8]. This priority aligns with several current initiatives to advance the mHealth evidence-base and its use in other sectors of health, particularly in LMICs [8].

## Health & risk factors in Armenia

**Fig. 1:**
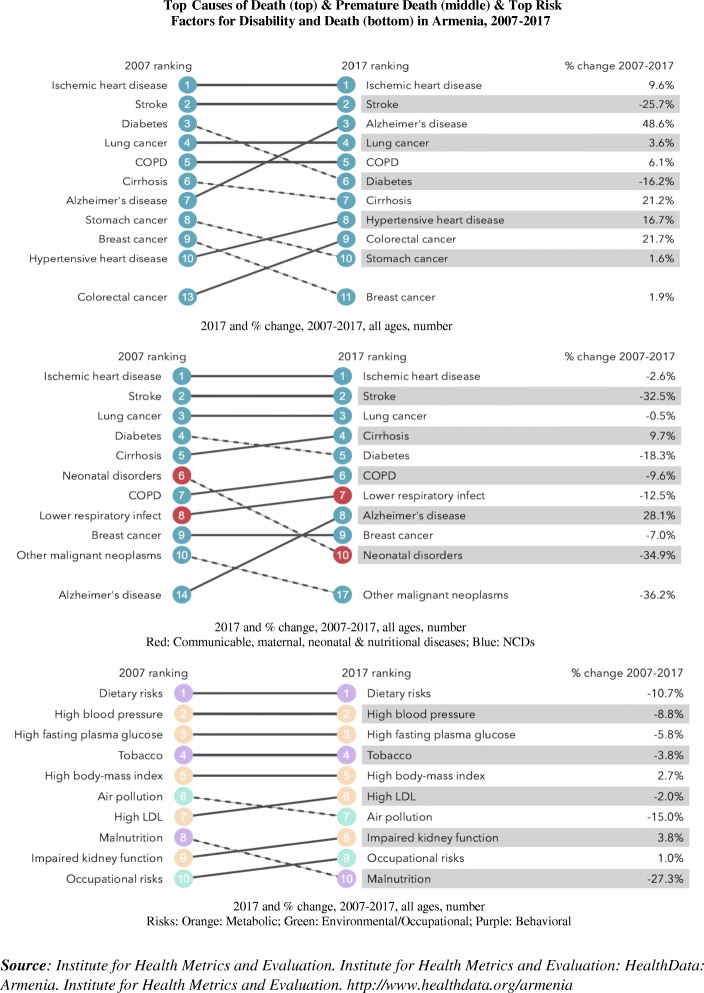
Top Causes of Death (top) & Premature Death (middle) & Top Risk. Factors for Disability and Death (bottom) in Armenia, 2007-2017

Armenia is in particular need of focused efforts to reduce its cancer burden. Despite a life expectancy of 76 years (72 in men vs. 79 in women; World Life Expectancy ranking of 87) [1–3], Armenia faces particular NCD-related challenges. Figure 1 [2] shows the top causes of death and premature death and the top risk factors for disability and death in Armenia in 2007–2017. Cancer is the second most prominent cause of death and premature death in Armenia [2]. Moreover, Armenia has the 2nd highest rate of cancer-related deaths in the world (198 per 100 K people; behind Mongolia) [2, 3]. While some of its neighbors rank closely behind (e.g., Hungary 3rd, Slovakia 5th, Russia 10th, etc.), its closest neighbors rank far better (Turkey 41st, Georgia 82nd, Azerbaijan 93rd, Iran 120th) [1–3]. Furthermore, Armenia has the 8th highest lung cancer death rate and also fairs poorly for other cancers (e.g., 3rd for pancreas, 7th bladder, 19th breast), despite Armenia ranking 97th in cancer prevalence worldwide [1–3].

Among the top risk factors in Armenia is tobacco use – which is associated with nearly all top causes of death and premature death, including cancer [2]. Overall, 28% of Armenian adults currently smoke (almost all smoke daily) [1]; noteworthy is that (while ~ 2% of women smoke) 52% of men smoke – the 11th highest smoking prevalence among men globally. Not surprisingly, SHSe in Armenia is high (e.g., > 50% report past-month SHSe at home; ~ 1/3 at work) [9], underscoring broad impact of tobacco in Armenia. Furthermore, Armenia’s health system is evolving with regard to infrastructure for NCD prevention and control, including CPC, making enhancements to such infrastructure (such as those possible via mHealth) among the highest public health and healthcare priorities in Armenia [2].

## Addressing cancer and tobacco in Armenia

Armenia has identified gaps in implementing WHO-recommended cost-effective NCD preventive and clinical interventions. Several policy and legislative frameworks are in place for NCDs, including the 1) *National Strategic Program,* with focus on 3 diseases with high mortality rates (cardiovascular disease, cancer, diabetes); 2) *Strategic Program for the Prevention and Control of NCDs*; 3) *Strategy for Promoting Healthy Lifestyles*; and 4) *Tobacco Control Strategy*. Two priorities identified across these frameworks are 1) tobacco control and 2) technological advances to enhance healthcare access, quality, and cost-effectiveness.

### Tobacco control

Despite Armenia ratifying of the FCTC in 2004, tobacco control has lagged, and tobacco use and related diseases have shown little decline. However, in February 2020, Parliament passed new tobacco control legislation, which was signed into law in March 2020. This law harmonizes Armenian tobacco control with the WHO FCTC and takes progressive action to extend smoke-free bans apply to all tobacco products and to all public places and toward industry marketing (i.e., tobacco display/ad bans, plain packaging). This provides a pivotal time for 1) catalyzing legislation impact to reduce tobacco use, morbidity, and mortality and 2) advancing the global evidence-base for tobacco control by researching the implementation and impact of the legislation.

### mHealth in Armenia

Mobile phone and internet access and utilization have advanced considerably throughout the country in both rural and urban settings. In the context of this broad coverage, some advances have been made. For example, in 2014, an initiative to institutionalize patient-centered tuberculosis treatment and improve treatment adherence was launched, led by the Turpanjian School of Public Health at American University of Armenia, the Ministry of Health of the Republic of Armenia, and the National Center of Pulmonology. This multifaceted program is now implemented nationwide and includes text message reminders to prompt patients to take their medication and track treatment adherence. In addition, Armenia’s *Strategy for the Development of Science* focuses on developing its technology and healthcare IT infrastructures, making this a pivotal time to bolster mHealth as potentially high-impact asset that can be leveraged to reduce cancer risk behaviors, reduce health system costs, improve access to healthcare services, and/or improve quality and effectiveness of healthcare services. However, Armenia has yet to fully take advantage of mHealth or telemedicine approaches for CPC, underscoring the need for training programs that promote mHealth in the context of NCD treatment and research.

## Need for research training in CPC risk factors

Despite significant progress in enhancing research capacity in Armenia over the past decade, additional efforts are needed, particularly in CPC-related research. Public health training programs remain underdeveloped in the area of CPC, and in-country research expertise regarding CPC and related areas (e.g., mHealth, policy) is relatively limited, particularly given the need to address the diverse and complex determinants of cancer onset, prevention, and management. Moreover, critical gaps in research dissemination and knowledge translation from evidence to policy and practice continue to exist. Thus, there is a need to continue to enhance public health infrastructure, in-country CPC leaders across various relevant disciplines must be further developed and supported, and medical and public health training must more fully integrate CPC and research dissemination and translation to inform policy and practice and ultimately reduce Armenia’s cancer burden [10].

## Data Availability

N/A.
